# Bacterial co-infections in community-acquired pneumonia caused by SARS-CoV-2, influenza virus and respiratory syncytial virus

**DOI:** 10.1186/s12879-022-07089-9

**Published:** 2022-01-31

**Authors:** Pontus Hedberg, Niclas Johansson, Anders Ternhag, Lina Abdel-Halim, Jonas Hedlund, Pontus Nauclér

**Affiliations:** 1 Department of Medicine Solna, Karolinska Institutet, 171 77 Stockholm, Sweden; 2 Department of Infectious Diseases, Karolinska University Hospital, Stockholm, Sweden

**Keywords:** SARS-CoV-2, COVID-19, Influenza, Respiratory syncytial virus, Co-infection

## Abstract

**Background:**

A mismatch between a widespread use of broad-spectrum antibiotic agents and a low prevalence of reported bacterial co-infections in patients with SARS-CoV-2 infections has been observed**.** Herein, we sought to characterize and compare bacterial co-infections at admission in hospitalized patients with SARS-CoV-2, influenza or respiratory syncytial virus (RSV) positive community-acquired pneumonia (CAP).

**Methods:**

A retrospective cohort study of bacterial co-infections at admission in SARS-CoV-2, influenza or RSV-positive adult patients with CAP admitted to Karolinska University Hospital in Stockholm, Sweden, from year 2011 to 2020. The prevalence of bacterial co-infections was investigated and compared between the three virus groups. In each virus group, length of stay, ICU-admission and 30-day mortality was compared in patients with and without bacterial co-infection, adjusting for age, sex and co-morbidities. In the SARS-CoV-2 group, risk factors for bacterial co-infection, were assessed using logistic regression models and creation of two scoring systems based on disease severity, age, co-morbidities and inflammatory markers with assessment of concordance statistics.

**Results:**

Compared to influenza and RSV, the bacterial co-infection testing frequency in SARS-CoV-2 was lower for all included test modalities. Four percent [46/1243 (95% CI 3–5)] of all SARS-CoV-2 patients had a bacterial co-infection at admission, whereas the proportion was 27% [209/775 (95% CI 24–30)] and 29% [69/242 (95% CI 23–35)] in influenza and RSV, respectively. *S. pneumoniae* and *S. aureus* constituted the most common bacterial findings for all three virus groups. Comparing SARS-CoV-2 positive patients with and without bacterial co-infection at admission, a relevant association could not be demonstrated nor excluded with regards to risk of ICU-admission (aHR 1.53, 95% CI 0.87–2.69) or 30-day mortality (aHR 1.28, 95% CI 0.66–2.46) in adjusted analyses. Bacterial co-infection was associated with increased inflammatory markers, but the diagnostic accuracy was not substantially different in a scoring system based on disease severity, age, co-morbidities and inflammatory parameters [C statistic 0.66 (95% CI 0.59–0.74)], compared to using disease severity, age and co-morbidities only [C statistic 0.63 (95% CI 0.56–0.70)].

**Conclusions:**

The prevalence of bacterial co-infections was significantly lower in patients with community-acquired SARS-CoV-2 positive pneumonia as compared to influenza and RSV positive pneumonia.

**Supplementary Information:**

The online version contains supplementary material available at 10.1186/s12879-022-07089-9.

## Background

Knowledge of pathogens causing community-acquired pneumonia (CAP) has impact on the selection of empirical antimicrobial treatment and on the prognosis of the patient [1, 2]. Respiratory viruses are recognized as an increasingly important cause of CAP in adults, with bacterial co-infections a common feature in CAP caused by influenza viruses and respiratory syncytial virus (RSV) [3–5].

The ability of severe acute respiratory syndrome corona virus 2 (SARS-CoV-2) to cause severe lower respiratory infections with a high morbidity and mortality is by now well established [6]. Yet, it is still not elucidated to which extent SARS-CoV-2 causes pneumonia by itself or by acting in conjunction with other respiratory bacterial pathogens. Although previous retrospective studies have described the incidence of bacterial co-pathogens in patients with SARS-CoV-2, no investigations have to our knowledge strictly investigated the concomitant occurrence of both SARS-CoV-2 and bacteria in patients admitted to hospital with confirmed CAP [7–10]. In two systematic reviews, most included studies have been conducted in China [Langford et al., 79% (19 of 24 studies) and Lansbury et al., 77% (23 of 30 studies)] [11, 12]. Further, the effect of bacterial co-infections on coronavirus disease 2019 (COVID-19) clinical outcomes is not well known [10, 13]. Data is limited on optimal co-infection testing strategies in COVID-19 and the utility of biomarkers such as C-reactive protein (CRP), white blood cell count (WBC), procalcitonin and neutrophil–lymphocyte ratio (NLR) to identify bacterial co-infections is conflicting [14–16].

The aim of this study was to investigate the prevalence of bacterial co-infections in patients with SARS-CoV-2 compared to influenza or RSV-positive community-acquired pneumonia at admission. Further, we aimed to compare co-infection testing rates and the use of antibiotics at admission in the three virus groups, as well as clinical outcomes in patients with and without a detected bacterial co-infection. Finally, the bacterial co-infection diagnostic accuracy of CRP, WBC, NLR and procalcitonin was assessed in SARS-CoV-2.

## Methods

### Patient population and study setting

We conducted a retrospective cohort study at Karolinska University Hospital, Stockholm, Sweden, an academic center with 1100 beds divided between two sites and serving a population of 2.3 million inhabitants. Adult patients (≥ 18 years), admitted through the emergency department between 2011-01-01 and 2020-12-31 with CAP and tested positive for SARS-CoV-2, influenza A (H3N2 and H1N1), influenza B (FluB) or RSV in respiratory samples at admission were reviewed for inclusion. CAP was defined as an increased body temperature (≥ 38 °C) or hypoxia (peripheral oxygen saturation < 95%) or tachypnea (respiratory rate > 20 breaths/min) and presence of new infiltrates on a chest radiograph or computed tomography documented within 48 h after admission. Patients with a previous positive test for the same respiratory virus (SARS-CoV-2, influenza or RSV) within 90 days and patients with a previous hospitalization at Karolinska University Hospital within the last 30 days were excluded.

The study was approved by the Swedish Ethical Review Authority (Dnr 2018/1030-31, COVID-19 research amendment Dnr 2020-01385). All methods were performed in accordance with the relevant guidelines and regulations as stated in the Declarations of Helsinki.

### Data sources and definitions

Data was obtained from a database of electronic health records of all patients admitted to Karolinska University Hospital between January 2010 and February 2021, including demographics, ICD-10 codes, radiology reports, laboratory findings, vital signs, microbiology, intensive care data and mortality data. The electronic health record system is updated daily with information from the national death registry. Specific individual comorbidities as well as Charlson Comorbidity Index (CCI) were based on ICD-10 codes recorded from up to five years before admission (Additional file 1: Table S1). Diagnoses and procedures in the immunosuppression group included HIV, certain types of solid and hematological malignancies, some diseases of blood and blood-forming organs, immune mechanism disorders, chronic kidney disease, radiotherapy and organ transplant status. As for vital signs and laboratory parameters, the worst value −24 h to +24 h from admission was used. Radiology reports from chest radiography and computer tomography performed within the first 48 h of admission was manually reviewed for presence of new infiltrates. Data on the following microbiological analyses performed 24 h before to 48 h after admission to hospital was collected; cultures from nasopharyngeal samples, lower respiratory tract (LRT: sputum, tracheal and bronchial secretions) and blood, PCR from respiratory tract secretions for *Mycoplasma pneumoniae, Chlamydophila pneumoniae* and *Chlamydophila psittaci*, sputum culture/or PCR for *Legionella pneumophila* and urine antigen detection for *Streptococcus pneumoniae* and *L. pneumophila*.

### Microbiological diagnostic criteria

Influenza virus A and B, RSV (A and B) and SARS-CoV-2 were detected from respiratory samples with real-time PCR (see Additional file 1: Table S2 for details). For nasopharyngeal cultures, growth of *S. pneumoniae* was considered a significant finding [17, 18]. For LRT cultures, a quantitative cutoff of ≥ 10^3^ colony-forming units per milliliter (CFU/mL) for protected brush specimens, ≥ 10^4^ CFU/mL for bronchoalveolar lavage (BAL) and ≥ 10^5^ CFU/mL for sputum and tracheal secretions were determined as significant [19]. Identification of *M. pneumoniae and C. pneumoniae* by PCR, detection of *L. pneumophila* by culture or PCR from sputum or positive urine antigen assay, and detection of *S. pneumoniae* in urine antigen *test,* were considered significant findings [20].

### Outcomes

The proportions of bacterial co-infections in the SARS-CoV-2, influenza and RSV-cohorts were investigated. Length of stay (LOS), ICU-admission and 30-day mortality was compared in patients with and without documented bacterial co-infections.

### Statistical analysis

Within each virus-specific cohort, sex, age-category and CCI-score category adjusted regression analyses were performed to compare outcomes in patients with and without detected bacterial co-infection. Hospital LOS was analyzed using Fine and Gray models, with in-hospital mortality being a competing event to discharge alive. Admission to the ICU and 30-day all-cause mortality was analyzed using Cox proportional hazards models. For SARS-CoV-2 positive patients, the association of baseline characteristics and a priori defined cut-offs for the four inflammatory markers CRP (< 50, 50–149, ≥ 150 mg/L), WBC (≤ 8.8, 8.9–12.0, > 12.0 × 10^9^ cells/L), procalcitonin (< 0.50, 0.50–1,99. ≥ 2.00) and NLR (< 10.0, 10.1–20.0, > 20.0) with bacterial co-infection was investigated using crude and age, sex, CCI and disease severity (CRB-65: confusion, respiratory rate ≥ 30, blood pressure systolic < 90 or diastolic ≤ 60 and age ≥ 65 years) adjusted logistic regression models. Further, in order to investigate the potentially added diagnostic accuracy measures of inflammatory markers, two scoring systems were defined based on the multivariate logistic regression models, with main emphasis on clinical applicability. In the first scoring system, one point was given per CRB-65 point (0–4 points) and one additional point was given for presence of any of the analyzed co-morbidities, thus ranging from 0 to 5 points. The second scoring system used the same variables and points as in the first scoring system, but also included 1 point for CRP ≥ 50 mg/L, 1 point for WBC > 12.0 × 10^9^ cells/L and 1 point for procalcitonin ≥ 2.00 μg/L, thus ranging from 0–8 points. Based on the distribution of the scores for all SARS-CoV-2 patients, three categories were defined for the first (0–1, 2–3 and 4–5 points) and second (0–2, 3–5 and > 5 points) scoring system. These score categories were then analyzed in logistic regression models, and the C statistic of the two models were compared, with 95% confidence intervals based on 2000 stratified bootstrap replicates.

To account for differential testing among the three virus groups, a sensitivity analysis was performed restricted to patients with a blood culture, NPH culture, LRT culture and urinary bacterial antigen testing performed.

All statistical analyses were performed in R version 4.0.3.

## Results

### Patient characteristics, admission status and clinical outcomes in the three virus groups

A total of 2260 healthcare episodes (1243 SARS-CoV-2, 775 influenza and 242 RSV) from 2238 patients were included in the study, see study flowchart with inclusion and exclusion criteria in Additional file 1: Fig. S1. The SARS-CoV-2 cohort was younger and more often male [median age 62 years (IQR 52–73), 65% male] as compared to influenza [median age 69 years (IQR 54–79), 52% male] and RSV [median age 71 years (IQR 61–81), 47% male] (Table 1). Diabetes was more common in the SARS-CoV-2 cohort as compared to influenza and RSV, whereas malignancy and immunosuppression were more common in the influenza and RSV cohorts as compared to SARS-CoV-2. The proportions of tachypnoea and reduced alertness were similar between all three virus groups, whereas a higher proportion of influenza and RSV patients had hypotension at admission. Thirty-three percent (407/1243) of SARS-CoV-2 patients received at least one dose of antibiotics outside the ICU at admission, whereas for influenza and RSV, the proportion was 84% (650/775) and 88% (213/242). Twenty-five percent (313/1243) of SARS-CoV-2 patients were admitted to the ICU, compared to 17% (131/775) and 16% (38/242) of influenza and RSV patients, respectively. Thirty-day mortality was 11% (142/1243), 7% (55/775) and 7% (16/242) for SARS-CoV-2, influenza and RSV, respectively.

**Table 1:** Patient characteristics, admission characteristics and clinical outcomes in the SARS-CoV-2, influenza and RSV cohorts

	SARS-CoV-2 (n = 1243)	Influenza (n = 775)	RSV (n = 242)
Patient characteristics			
Male sex, n (%)	805 (65)	403 (52)	113 (47)
Age, median (IQR), years	62 (52–73)	69 (54–79)	71 (61–81)
≥ 65, n (%)	520 (42)	442 (57)	170 (70)
CCI, median (IQR), points	1 (0–2)	1 (0–3)	2 (1–3)
0–1, n (%)	871 (70)	406 (52)	101 (42)
2–4, n (%)	291 (23)	279 (36)	103 (43)
≥ 5, n (%)	81 (7)	90 (12)	38 (16)
Specific comorbidities^a^			
Diabetes mellitus, n (%)	338 (27)	139 (18)	47 (19)
Hypertension, n (%)	565 (45)	265 (34)	95 (39)
Chronic cardiac disease, n (%)	375 (30)	266 (34)	101 (42)
Chronic respiratory disease, n (%)	444 (36)	233 (30)	82 (34)
Chronic kidney disease, n (%)	86 (7)	65 (8)	29 (12)
Malignancy, n (%)	110 (9)	167 (22)	75 (31)
Immunosuppression, n (%)	192 (15)	236 (30)	100 (41)
Any of the comorbidities above, n (%)	911 (73)	560 (72)	198 (82)
Admission characteristics			
Respiratory rate ≥ 30 breaths/min, n (%)	393 (32)	262 (34)	75 (31)
Systolic blood pressure < 90 mmHg or diastolic blood pressure ≤ 60 mmHg, n (%)	340 (28)	346 (45)	99 (42)
Non-alert, n (%)	117 (10)	55 (9)	15 (8)
Antibiotic administered at admission (outside the ICU), n (%)	407 (33)	650 (84)	213 (88)
Third-generation cephalosporins, n (%)	311 (25)	436 (56)	130 (54)
Penicillins, n (%)	73 (6)	246 (32)	83 (34)
Fluoroquinolones, n (%)	14 (1)	72 (9)	26 (11)
Clinical outcomes			
LOS, median (days)	8 (5–15)	5 (3–8)	5 (3–9)
ICU-admission, n (%)	313 (25)	131 (17)	38 (16)
30-day mortality, n (%)	142 (11)	55 (7)	16 (7)

^a^See Additional file 1: Table S1 for included ICD-10 codes per specific comorbidity

CCI, Charlson Comorbidity Index; IQR, Interquartile range; RSV, Respiratory syncytial virus; SARS-CoV-2, Severe acute respiratory syndrome coronavirus 2

### Bacterial co-infection testing frequency and positivity rate

**Fig. 1:**
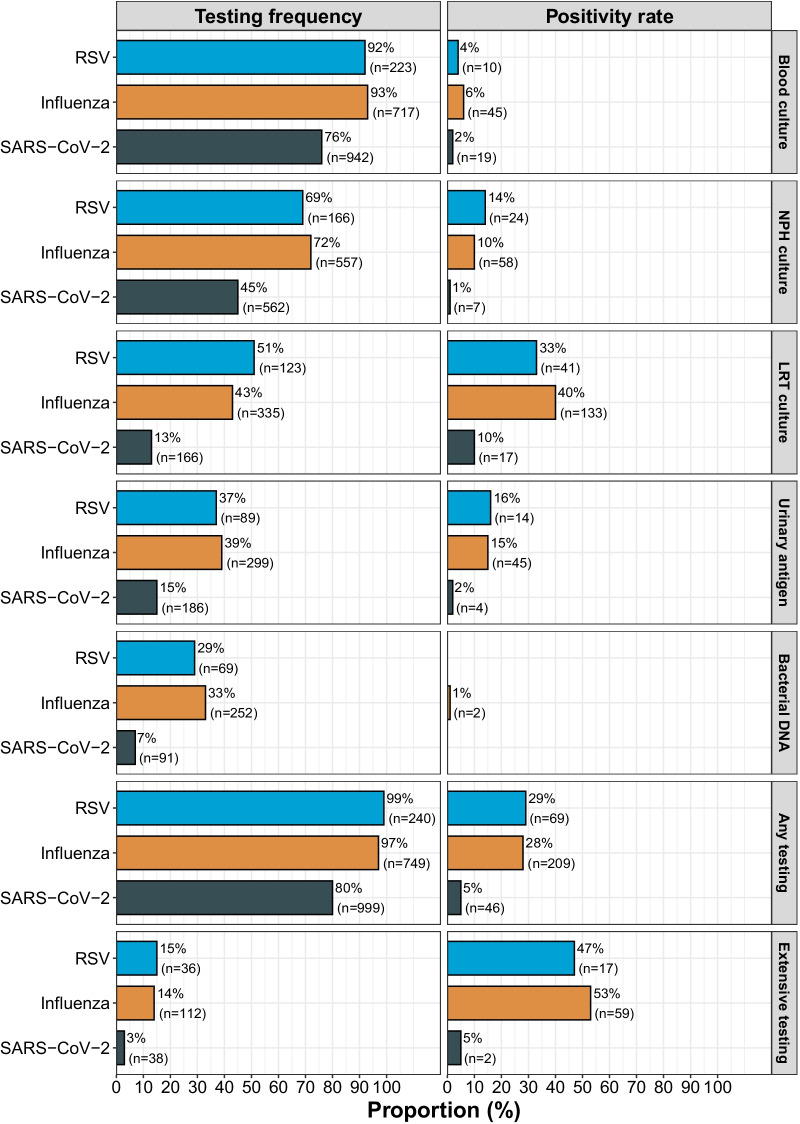
Bacterial co-infection testing frequency and positivity rate per virus group and diagnostic test modality. For testing frequency, the proportion represents the number of patients having each test performed at admission divided by the total number of patients per virus group. The number of tested individuals are found within brackets. For positivity rate, the proportion represents the number of patients with a positive test per test modalitiy divided by the total number of tested patients per virus group. The number of positive individuals are found within brackets. RSV, respiratory syncytial virus; SARS-COV-2,severe acute respiratory syndrome coronavirus 2; NPH, nasopharyngeal; LRT, lower respiratory tract

All bacterial co-infection testing was performed in a significantly lower proportion of SARS-CoV-2 patients compared to influenza and RSV (Fig. 1). This was in particular true for LRT cultures, urinary bacterial antigen tests and bacterial DNA tests, where a more than two-fold difference was observed. Three percent (38/1243) of the SARS-CoV-2 patients had a blood culture, NPH culture, LRT culture as well as urinary bacterial antigen testing performed (hereafter referred to as extensively tested) at admission. For influenza and RSV, 14% (112/775) and 15% (36/242) of the patients were extensively tested. No obvious difference in testing strategies were observed between first and second wave for SARS-CoV-2 patients (Additional file 1: Fig. S2).

Four percent [46/1243 (95% CI 3–5)] of all SARS-CoV-2 patients had a bacterial co-pathogen diagnosed at admission, whereas the proportion was 27% [209/775 (95% CI 24–30)] and 29% [69/242 (95% CI 23–35)] in influenza and RSV. Similar findings were observed when excluding *S. pneumoniae* positive nasopharyngeal cultures, with the proportion of bacterial co-infections being 3% [40/1243 (95% CI 2–4)] in SARS-CoV-2, 24% [188/775 (95% CI 21–27)] in influenza and 24% [57/242 (95% CI 19–29)] in RSV. When restricting the analysis to only extensively tested patients, the proportion of bacterial co-infections was still lower in SARS-CoV-2 [5%, 2/38 (95% CI 1–17)] as compared to influenza [53%, 59/112 (95% CI 43–62)] and RSV [47%, 17/36 (95% CI 32–63)]. LRT culture was the testing modality with highest positivity rate for SARS-CoV-2, influenza as well as RSV (10%, 40% and 33%, respectively).

**Fig. 2:**
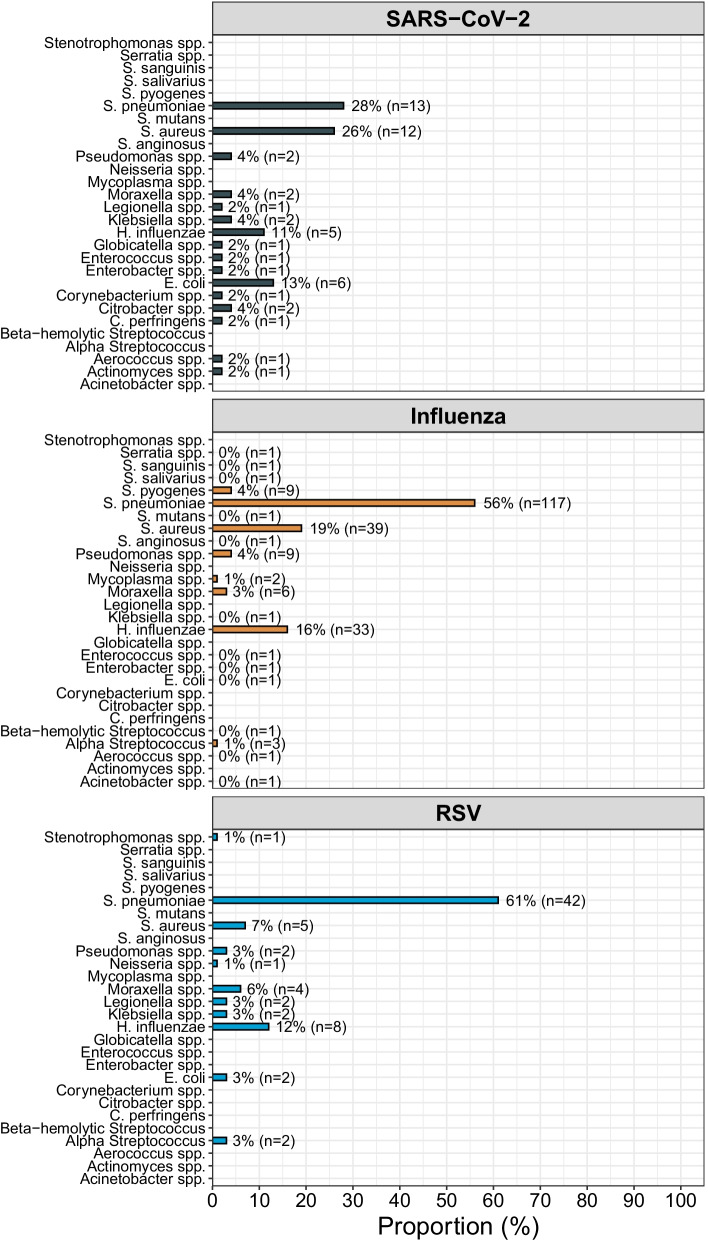
Bacterial co-infection etiologies in SARS-CoV-2, influenza and RSV. Bacterial etiologies in SARS-CoV-2 (upper), influenza (middle) and RSV (lower). The proportion represents the proportion of all patients tested positive per virus category (SARS-CoV-2 = 46, Influenza = 209, RSV = 69). RSV, respiratory syncytial virus; SARS-COV-2,severe acute respiratory syndrome coronavirus 2, spp, species

The most common bacterial agent was *S. pneumoniae* for SARS-CoV-2 (28%, 13/46), influenza (56%, 117/209) and RSV (61%, 42/69) (Fig. 2). Excluding nasopharyngeal findings the corresponding figures were 18% (7/40), 51% (95/188), and 53% (30/57) respectively. In SARS-CoV-2, *S. aureus* (26%, 12/46), *E. coli* (13%, 6/46) and *H. influenzae* (11%, 5/46) were the most frequent findings after *S. pneumoniae*. A complete description of bacterial findings in blood and LRT cultures is found in Additional file 1: Fig. S3.

### Outcomes in patients with and without bacterial co-infection

The LOS for patients with and without a bacterial co-infection in SARS-CoV-2 was 7 days (IQR 4–18) and 8 days (5–15), as compared to 5 days (IQR 3–10) and 4 days (IQR 3–7) in influenza and 5 days (IQR 3–7) and 5 days (IQR 3–9) in RSV, respectively (Table 2). In age, sex, and comorbidity-adjusted competing risk analyses, with in-hospital mortality as a competing event, no significant difference could be observed in being discharged alive between patients with and without a bacterial co-infection in SARS-CoV-2 and RSV. For influenza patients with a bacterial co-infection, the adjusted subdistribution hazard ratio for being discharged alive was 0.76 (95% CI 0.64–0.90) as compared to patients without a detected bacterial co-infection. Twenty-eight percent (13/46) of SARS-CoV-2 patients with a bacterial co-infection was admitted to the ICU, compared to 25% (300/1197) for patients without a bacterial co-infection. For all three virus group, no significant difference was observed for patients with a bacterial co-infection to get admitted to the ICU compared to patients without a bacterial co-infection. The 30-day all cause mortality was 22% (10/46) in SARS-CoV-2 patients with a bacterial co-infection as compared to 11% (132/1197) in patients without findings of a bacterial co-pathogen [aHR 1.28 (95% CI 0.66–2.46)]. For influenza, the corresponding figures were 5% (10/209) vs. 8% (45/566), and for RSV 1% (1/69) vs. 9% (15/173), respectively. For all three viruses, a significant effect of detected bacterial co-infection on 30-day all-cause mortality could neither be demonstrated nor excluded.

**Table 2:** Outcomes with and without bacterial co-infection in SARS-CoV-2, influenza and RSV

Outcome	SARS-CoV-2			Influenza			RSV		
	No co-infection (n = 1197)	Co-infection (n = 46)	Adjusted ratio (95% CI)^a^	No co-infection (n = 566)	Co-infection (n = 209)	Adjusted ratio (95% CI)^a^	No co-infection (n = 173)	Co-infection (n = 69)	Adjusted ratio (95% CI)^a^
Length of stay, median (IQR), days^b^	8 (5–15)	7 (4–18)	0.82 (0.54–1.23)	4 (3–7)	5 (3–10)	0.76 (0.64–0.90)	5 (3–9)	5 (3–7)	1.16 (0.86–1.56)
ICU-admission, n (%)^c^	300 (25)	13 (28)	1.53 (0.87–2.69)	79 (14)	52 (25)	1.05 (0.73–1.50)	30 (17)	8 (12)	1.41 (0.58–3.47)
30-day mortality^c^	132 (11)	10 (22)	1.28 (0.66–2.46)	45 (8)	10 (5)	0.72 (0.36–1.44)	15 (9)	1 (1)	0.21 (0.03–1.57)

CI, Confidence interval; ICU, Intensive care unit; IQR, Interquartile range; LRT, Lower respiratory tract; NPH, Nasoparyngeal; RSV, Respiratory syncytial virus; SARS-CoV-2, Severe acute respiratory syndrome coronavirus 2

^a^Adjusted for age-category, sex and Charlson comorbidity index category

^b^Analyzed by a Fine and Gray model, with presented adjusted subdistribution hazard ratios

^c^Analyzed by a Cox proportional hazards model, with presented adjusted hazard ratios

### Discriminating bacterial co-infections in SARS-CoV-2 CAP

SARS-CoV-2 patients with a detected bacterial co-infection were older [median age 70 years (IQR 51–78)] as compared to patients without a bacterial co-infection [median age 62 years (IQR 52–73)] [OR 2.03 (95% CI 1.12–3.74)] (Table 3). Further, they more often had underlying chronic cardiac diseases [52% (24/46)] and immunosuppression [28% (13/46)] as compared to patients without a bacterial co-infection [29% (351/1197) and 15% (179/1197), respectively]. A detected bacterial co-infection was associated with an increased risk of a CRB-65 ≥ 3 points, OR 2.72 (95% CI 1.25–5.43).

**Table 3:** Risk factors for bacterial co-infections in SARS-CoV-2 community-acquired pneumonia

	No co-infection (n = 1197)	Co-infection (n = 46)	OR (95% CI)	aOR (95% CI)^a^
Baseline characteristics				
Male sex, n (%)	776 (65)	29 (63)	0.93 (0.51–1.74)	–
Age, median (IQR), years	62 (52–73)	70 (51–78)	–	–
≥ 65, n (%)	493 (41)	27 (59)	2.03 (1.12–3.74)	–
CCI, median (IQR), points	1 (0–2)	1 (1–3)	–	–
0–1, n (%)	843 (70)	28 (61)	Ref	–
2–4, n (%)	281 (23)	10 (22)	1.07 (0.49–2.16)	–
≥ 5, n (%)	73 (6)	8 (17)	3.30 (1.36–7.19)	–
Specific comorbidities				
Diabetes mellitus, n (%)	323 (27)	15 (33)	1.31 (0.68–2.42)	–
Hypertension, n (%)	539 (45)	26 (57)	1.59 (0.88–2.91)	–
Chronic cardiac disease, n (%)	351 (29)	24 (52)	2.63 (1.45–4.78)	–
Chronic respiratory disease, n (%)	422 (35)	22 (48)	1.68 (0.93–3.04)	–
Chronic kidney disease, n (%)	80 (7)	6 (13)	2.09 (0.78–4.74)	–
Malignancy, n (%)	103 (9)	7 (15)	1.91 (0.76–4.12)	–
Immunosuppression, n (%)	179 (15)	13 (28)	2.24 (1.12–4.24)	–
Any of the comorbidities above, n (%)	868 (73)	43 (93)	5.44 (1.96–22.52)	–
Severity scores				
CRB-65, median (IQR), points	1 (0–2)	1 (1–2)	–	–
3–4	111 (9)	10 (22)	2.72 (1.25–5.43)	–
Admission values				
CRP, median (IQR), mg/L^b^	97 (50–167)	121 (93–173)	–	–
< 50	289 (24)	3 (7)	Ref	Ref
50–149	574 (46)	28 (61)	4.93 (1.73–20.74)	4.82 (1.68–20.34)
≥ 150	360 (30)	15 (33)	4.01 (1.31–17.45)	3.92 (1.27–17.11)
WBC, median (IQR), 10^9^ cells/L^c^	6.9 (5.2–9.4)	9.6 (7.1–13.4)	–	–
≤ 8.8	851 (71)	22 (48)	Ref	Ref
8.9–12.0	213 (18)	8 (17)	1.45 (0.60–3.18)	1.34 (0.55–2.97)
> 12.0	132 (11)	16 (35)	4.69 (2.36–9.11)	4.27 (2.12–8.42)
Procalcitonin, median (IQR), μg/L^d^	0.17 (0.10–0.40)	0.89 (0.15–6.50)	–	–
< 0.50	819 (79)	19 (46)	Ref	Ref
0.50–1.99	156 (15)	9 (22)	2.49 (1.05–5.45)	2.28 (0.96–5.06)
≥ 2.00	61 (6)	13 (32)	9.19 (4.25–19.36)	8.01 (3.46–18.06)
NLR, median (IQR), ratio^e^	4.7 (2.9–8.1)	6.6 (4.4–10.5)	–	–
≤ 10.0	893 (84)	28 (68)	Ref	Ref
10.1–20.0	146 (14)	8 (20)	1.75 (0.73–3.74)	1.49 (0.61–3.25)
> 20.0	29 (3)	5 (12)	5.50 (1.77–14.22)	4.90 (1.53–13.15)

aOR, Adjusted odds ratio; CCI, Charlson Comorbidity Index; CI, Confidence interval; CRB-65, Confusion-Respiration-Blood pressure > 65 years; CRP, C-reactive protein; NLR, Neutrophil–Lymphocyte ratio; OR, Odds ratio; Ref, Reference; WBC, White blood cell count

^a^Adjusted for age-category, CCI-category and CRB-65 category

^b^No admission value recorded for 1 health care episode

^c^No admission value recorded for 1 health care episode

^d^No admission value recorded for 166 health care episodes

^e^No admission value recorded for 134 health care episodes

All investigated inflammatory markers, CRP, WBC, procalcitonin and NLR, were elevated in patients with a detected bacterial co-infection as compared to patients without a bacterial co-infection. After adjusting for age, sex, CCI and pneumonia severity, bacterial co-infection was associated with a CRP ≥ 50 mg/L [CRP 50–149: aOR 4.82 (95% CI 1.68–20.34), CRP ≥ 150: aOR 3.92 (95% CI 1.27–17.11)], WBC > 12.0 × 10^9^ cells/L [aOR 4.27 (95% CI 2.12–8.42)], procalcitonin ≥ 2.00 [aOR 8.01 (95% CI 3.46–18.06)] and NLR > 20.0 [aOR 4.90 (95% CI 1.53–13.15)].

In the scoring system based on CRB-65 and presence of any co-morbidity, 2% (10/512) of patients with a score of 0–1 points had a bacterial co-infection, compared to 4% (26/615) for patients with 2–3 points and 9% (10/116) for patients with 4–5 points. The C statistic for the model based on these three categories was 0.63 (95% CI 0.56–0.70). In the scoring system also including binary cut-offs for CRP (≥ 50 mg/L), WBC (> 12.0 × 10^9^ cells/L) and procalcitonin (≥ 2.00 μg/L), 2% (8/451) of patients with a score of 0–2 points had a bacterial co-infection, compared to 4% (24/573) for patients with a score of 3–5 points and 17% (9/53) for patients with a score of 6–8 points. The C statistic for the model based on these three categories was 0.66 (95% CI 0.59–0.74).

## Discussion

In this observational investigation where we strictly studied adult patients admitted to hospital with CAP and positive for either SARS-CoV-2, influenza or RSV, the most important findings were: (1) Compared to influenza and RSV, the bacterial co-infection testing frequency in SARS-CoV-2 was lower for all included test modalities; (2) the bacterial co-infection rate was low in the SARS-CoV-2 cohort and significantly lower as compared to both influenza and RSV; (3) although *S. pneumoniae* was the most common bacterial co-pathogen detected in all the three viral cohorts studied, it was relatively less common compared to other pathogens in SARS-CoV-2 CAP; (4) no significant differences were observed regarding LOS, ICU-admission, as well as 30-day mortality in SARS-CoV-2 positive patients with vs. without a diagnosed bacterial co-infection; (5) Bacterial co-infection was associated with increased inflammatory markers, but the diagnostic accuracy was not significantly increased in a scoring system based on disease severity, age, co-morbidities and inflammatory parameters [C statistic 0.66 (95% CI 0.59–0.74)], compared to using disease severity, age and co-morbidities only [C statistic 0.63 (95% CI 0.56–0.70)].

CAP is a common disease responsible for substantial morbidity and mortality among adults [5]. It is by now known that many different respiratory viruses seem to act in conjunction with bacteria for development of CAP [21]. Herein we found a noticeable low prevalence of bacterial co-pathogens in SARS-CoV-2 patients and the yield was substantially lower compared to the cohorts with influenza and RSV. We also found the microbiological testing rates to be significantly lower for SARS-CoV-2 compared to influenza and RSV. The reasons for this are unknown but likely include concerns for viral transmission to medical staff during testing as well shortage of labor due to the excessive number of covid-19 patients admitted to hospital, especially in the beginning of the pandemic. However, even in the subgroup of patients undergoing extensive bacteriological testing, i.e. blood culture, NPH culture, LRT culture and urinary antigen testing, the proportion of diagnosed bacterial co-pathogens were still very low and significantly lower in SARS-CoV-2 (5%), compared to influenza (53%) and RSV (57%), supporting the results of a low prevalence of bacterial co-infections in Covid-19 patients at admission to hospital. We found no other studies that investigated the prevalence of bacterial co-infections in community-acquired SARS-CoV-2 pneumonia. When comparing our data with etiological studies including different kinds of SARS-CoV-2 respiratory tract infections from different clinical settings our results corroborate a low prevalence of bacterial co-infections. In a meta-analysis and systematic review where 24 studies with laboratory-confirmed SARS-CoV-2 infection across all healthcare settings were included, 3.5% of patients were reported to have a diagnosed co-infection [12]. Further, a recent retrospective cohort study in a Spanish university centre, and two UK secondary-care hospitals reported bacterial co-infections in 3.1% (31/989) and 3.2% (27/989) of patients with COVID-19 [7, 10]. Interestingly, *E. coli* was one of the most common detected pathogens together with *S. pneumoniae*. *S. aureus* and *H. influenzae.*

The administration of antibiotics at admission to patients with COVID-19 in this study was low, 33%, compared to 72% in previous literature reviews [8, 12]. Reported differences in the use of antibiotics could be due to several reasons, including assessment of different administration time intervals, different regional and national antibiotic policies, inclusion of antibiotics administered at the ICU as well as different phases of the SARS-CoV-2 pandemic.

The use of inflammatory markers to rule out bacterial co-infections has previously been investigated in a retrospective cohort study of 106 community-acquired pneumonia and 619 COVID-19 patients from the UK [14], reporting that a multivariate logistic regression model including baseline WBC and dynamics in CRP discriminated community-acquired pneumonia from COVID-19 with AUC 0.88 (95% CI 0.83–0.94). Herein, we investigated the association of CRP, WBC, procalcitonin and NLR with a detected bacterial co-infection. Our results corroborated the association of increased WBC at admission with bacterial co-infection, and further demonstrated an independent association for all four inflammatory markers when adjusting for age, CCI-category and pneumonia severity using CRB-65. Future prospective studies are needed to investigate whether these inflammatory markers can serve useful for clinical management and antibiotics administration in COVID-19.

Strengths of this study include the large study cohorts with the same strict inclusion criteria for all three virus groups, data covering both the first and second wave of the pandemic. Limitations are the retrospective study design performed in a two-hospital academic center, with inconsistent microbiological testing intensity in different patients. The lower testing frequency for all testing modalities in SARS-CoV-2 compared to influenza and RSV might lead to the prevalence of bacterial co-infection being more underestimated than in the other groups, thus overestimating the difference between the three virus cohorts. Further, the differences in testing frequency per test modality and virus group were different, with for instance LRT cultures being performed substantially more often in influenza and RSV. This has probably not only affected the estimated prevalence of bacterial co-infections, but also the etiological distribution. We decided not to use multiple imputation as we considered it likely that the microbiological test data were missing not at random. As such, we only performed a sensitivity analysis restricting the cohorts to individuals extensively tested. Further, a larger proportion of SARS-CoV-2 positive patients did not undergo a thoracic radiology (32%) as compared to influenza (16%) and RSV (8%). This might have resulted in bias of the SARS-CoV-2 cohort, with exclusion of individuals with potentially milder course of the disease where radiology was not performed, potentially affecting the external validity of our findings. As individuals with a detected bacterial co-infection presented with increased disease severity according to CRB-65, the proportion of bacterial co-infection might be even lower when thoracic radiology has been performed in a higher proportion of patients. Finally, we could not rule out preceding recent hospitalizations at other hospitals or pre-hospital antibiotic usage, possibly influencing the observed prevalence of co-infections as well as distribution of identified bacteria.

## Conclusions

The prevalence of bacterial co-infections in patients with CAP caused by SARS-CoV-2 was low compared to influenza and RSV, implying that antibiotic treatment seldom is necessary. However, when antimicrobial treatment is indicated it should be effective against *S. pneumoniae* and *S. aureus,* and possibly also against gram-negative bacteria. In neither of the three viruses, a significant effect of bacterial co-infection on risk of ICU-admission or 30-day mortality was observed. However, given the low numbers of detected bacterial co-infections in patients with SARS-CoV-2, resulting in wide confidence intervals, a relevant association could not be demonstrated nor excluded. Based on our characterization of positivity rate in relation to testing rates, it seems as a sensible use of resources not to test everyone hospitalized with COVID-19 for bacterial co-infections. Future prospective studies are warranted to further understand if bacterial co-infections in SARS-CoV-2 patients can be accurately predicted.

## Supplementary Information

**Additional file 1: Figure S1.** Flowchart of health care episodes included in the study. **Table S1.** ICD-10 codes for specific comorbidities. **Table S2.** Definition of positive microbiological tests. **Figure S2.** Bacterial co-infection testing practices over time in SARS-CoV-2 patients. **Figure S3.** Detected bacterial co-pathogens in LRT and blood cultures in SARS-CoV-2, influenza and RSV.

## Data Availability

No data are available. Data from deidentified electronic health records are not freely available due to protection of the personal integrity of the participants.

## Abbreviations

BAL: Bronchoalveolar lavage
CAP: Community-acquired pneumonia
CCI: Charlson Comorbidity Index
CFU: Colony-forming units
COVID-19: Coronavirus disease 2019
CRP: C-reactive protein
HCE: Healthcare episode
ICU: Intensive care unit
LOS: Length of stay
LRT: Lower respiratory tract
NLR: Neutrophil–lymphocyte ratio
RSV: Respiratory syncytial virus
SARS-CoV-2: Severe acute respiratory syndrome coronavirus 2
WBC: White blood cell count
