# Supplementary material for: Bacterial co-infections in community-acquired pneumonia caused by SARS-CoV-2, influenza virus and respiratory syncytial virus
Source: BMC Infect Dis. 2022 Jan 31;22:108. doi: 10.1186/s12879-022-07089-9 (PMC8802536; doi:10.1186/s12879-022-07089-9)
Supplement: Supplementary file 1 — Additional file 1: Figure S1. Flowchart of health care episodes included in the study. Table S1. ICD-10 codes for specific comorbidities. Table S2. Definition of positive microbiological tests. Figure S2. Bacterial co-infection testing practices over time in SARS-CoV-2 patients. Figure S3. Detected bacterial co-pathogens in LRT and blood cultures in SARS-CoV-2, influenza and RSV. [file 12879_2022_7089_MOESM1_ESM.docx]

**Supplementary material**

Bacterial co-infections in community-acquired pneumonia caused by SARS-CoV-2, Influenza virus and Respiratory Syncytial virus.

*Pontus Hedberg, Niclas Johansson, Anders Ternhag, Lina Abdel-Halim, Jonas Hedlund, Pontus Nauclér*

Table of Contents

[Figure S1. Flowchart of health care episodes included in the study. 2](#_Toc88722427)

[Table S1. ICD-10 codes for specific comorbidities 3](#_Toc88722428)

[Table S2. Definition of positive microbiological tests 4](#_Toc88722429)

[Figure S2. Bacterial co-infection testing practices over time in SARS-CoV-2 patients 5](#_Toc88722430)

[Figure S3. Detected bacterial co-pathogens in LRT and blood cultures in SARS-CoV-2, influenza and RSV 6](#_Toc88722431)

#

# Figure S1. Flowchart of health care episodes included in the study.

^
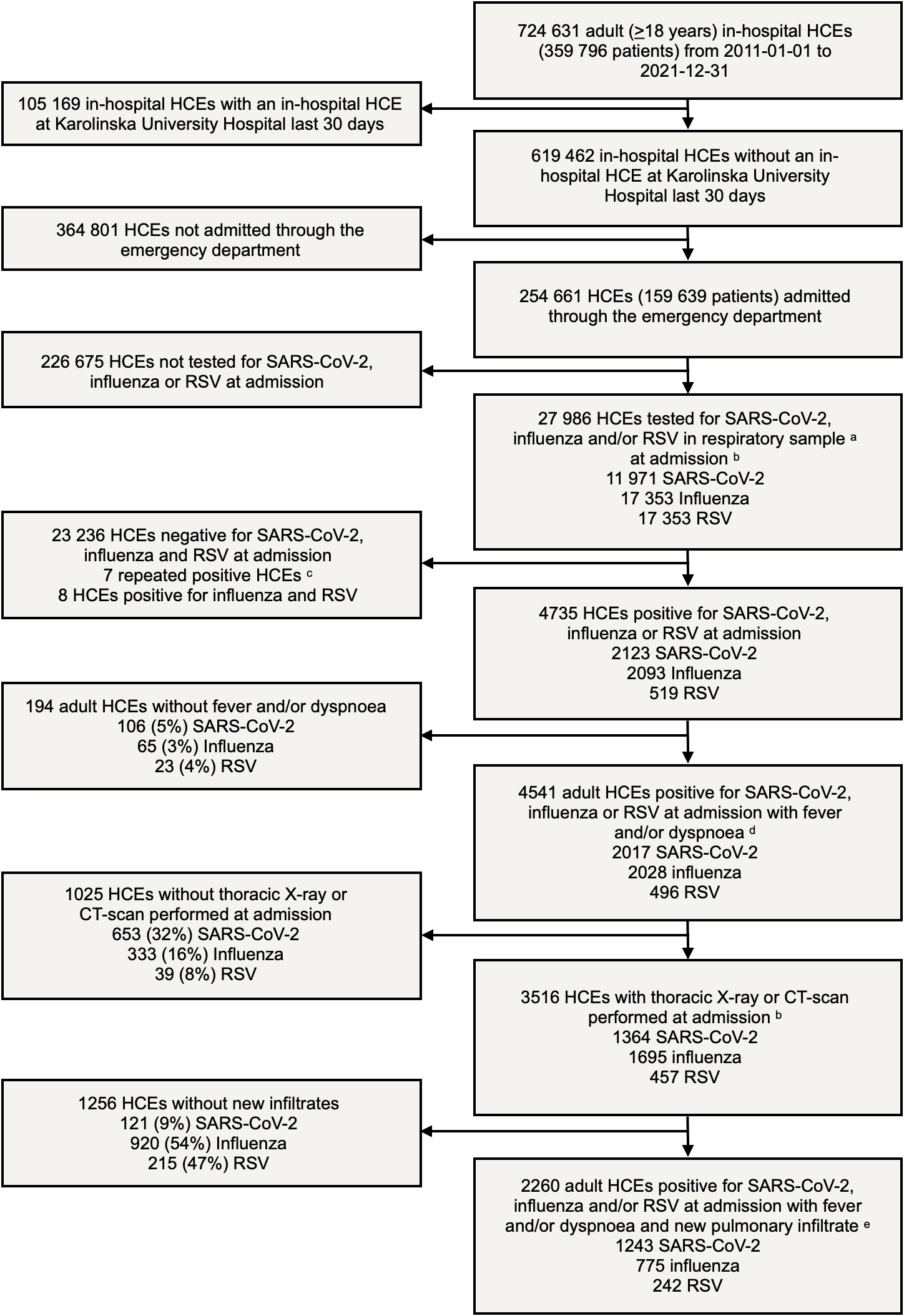
^

^a^ Nasopharynx, throat, sputum, tracheal secretion or bronchoalveolar lavage

^b^ Minus one day to plus two days from admission date

^c^ Tested positive for same pathogen within previous 90 days

^d^ Body temperature ≥38°C, oxygen saturation <95% or respiratory rate >20

^e^ New infiltrates that were not assessed as non-infectious or of uncertain significance according to radiologist.

**Abbreviations:** CT = Computed tomography, HCE = Health care episode, RSV = Respiratory syncytial virus, SARS-CoV-2 = Severe acute respiratory syndrome coronavirus 2

# Table S1. ICD-10 codes for specific comorbidities

| **Comorbidity category** | **ICD-10 codes ^a^** |
| --- | --- |
| Diabetes mellitus | E10-E14 |
| Hypertension | I10-I15 |
| Cardiac disease | I05-I08, I20-I22, I24-I28, I34-I37, I42, I44 -I50 |
| Chronic respiratory disease | J43-J47, J6-J7, J80-J84, J92-J96, J98-J99 |
| Chronic kidney failure | N18-N19 |
| Malignancy | C |
| Immunosuppression | B2, C0-C2, C30-C34, C37-C39, C4-C8, C90-C96, D70-D73, D80-D84, N18, Z21, Z51.0, Z51.1, Z94.0, Z94.1, Z94.2, Z94.3, Z94.4, Z94.6, Z94.8, Z94.9 |

^a^ The shortest text-pattern for identification of included ICD-10 codes are listed

# Table S2. Definition of positive microbiological tests

| **Test** | **Definition of positive test** | **Definition of negative test/Identified organisms considered non-significant/contaminants** |
| --- | --- | --- |
| SARS-CoV-2 | Confirmed positive for SARS-CoV-2 RNA | Negative or suspected positive for SARS-CoV-2 RNA |
| Influenza ^a^ | Confirmed positive for influenza A (H3N2 or H1N1) or influenza B (FluB) | Negative for influenza A or B RNA |
| RSV ^a^ | Confirmed positive for respiratory syncytial virus RNA | Negative for respiratory syncytial virus RNA |
| NPH bacterial culture | Growth of *S. pneumoniae* | Beta-hemolytic *Streptococci, Citrobacter spp., E. coli, Enterobacter spp., H. influenza, Klebsiella spp., Moraxella spp., Neisseria spp., Proteus spp., Pseudomonas spp., S. agalactiae, S. aureus, S. mitis, S. pyogenes, Serratia spp., Stenotrophomonas spp.,* Yeast |
| LRT culture | Brush specimens: >10^3^ CFU/mL  Bronchoalveolar lavage: >10^4^ CFU/mL  Sputum and tracheal secretions: >10^5^ CFU/ml | Non-quantitative cultures  Quantitative cultures lower than CFU threshold  Growth of Fungi |
| Blood culture | Growth of *Actinomyces spp., Actinotignum spp., Aerococcus spp., Bacteroides spp., Brevibacterium spp., C. perfringens, Citrobacter spp., E. Coli, Enterobacter spp., Enterococcus spp., Globicatella spp., Haemophilus spp., Klebsiella spp., Moraxella spp., Pseudomonas spp., Rothia spp., S. aureus, Streptococci spp.* | *Coagulase-negative Staphylococci, Corynebacterium spp., Micrococcus spp., Propionibacterium spp.* |
| Urinary bacterial antigen | Positive for *L. pneumophila* or *S. pneumoniae* | Negative for *L. pneumophila* and *S. pneumoniae* |
| Bacterial DNA tests | Positive for Chlamydophila DNA, Mycoplasma DNA or Legionella DNA | Negative for Chlamydophila DNA, Mycoplasma DNA and Legionella DNA |

a Diagnostics for a narrow respiratory virus panel including influenza A virus (IAV), influenza B virus (IBV), and respiratory syncytial virus (RSV) were performed using in-house realtime PCR assays until 14 September 2014 when they were replaced by the Simplexa Flu A/B \& RSV Kit (Focus Diagnostics Inc., Cypress (CA, USA)), which were in turn replaced by the Xpert Flu/RSV ((Cepheid, Solna, Sweden) from 15 May 2019 (*Tiveljung-Lindell A, Rotzén-Ostlund M, Gupta S, et al. Development and implementation of a molecular diagnostic platform for daily rapid detection of 15 respiratory viruses. J Med Virol. 2009;81(1):167-175. doi:10.1002/jmv.21368*).
 **Abbreviations:** CFU = Colony-forming units, LRT = Lower respiratory tract, NPH = Nasopharyngeal

# Figure S2. Bacterial co-infection testing practices over time in SARS-CoV-2 patients

#

# Note: Proportion of SARS-CoV-2 patients with test performed at admission per test modality. The first wave included all admissions before October 1, 2020 and the second wave included all admissions from October 1, 2020 and onwards.

**Abbreviations:** SARS-COV-2,severe acute respiratory syndrome coronavirus 2; NPH, nasopharyngeal; LRT, lower respiratory tract

# Figure S3. Detected bacterial co-pathogens in LRT and blood cultures in SARS-CoV-2, influenza and RSV

**Note:** Bacterial etiologies in positive blood cultures (upper panel) and lower respiratory tract samples (lower panel) by virus group. The proportion represents the proportion of all positive tests per virus category.

**Abbreviations:** SARS-COV-2, severe acute respiratory syndrome coronavirus 2; RSV, respiratory syncytial virus; LRT, lower respiratory tract
